# Systematic review of rehabilitation programmes initiated within 90 days of a transient ischaemic attack or ‘minor’ stroke: a protocol

**DOI:** 10.1136/bmjopen-2015-007849

**Published:** 2015-06-18

**Authors:** Neil Heron, Frank Kee, Michael Donnelly, Margaret E Cupples

**Affiliations:** 1 Centre for Public Health, School of Medicine, Dentistry and Biomedical Science, Queens University Belfast, Institute of Clinical Science B, Royal Victoria Hospital, Belfast, Antrim, UK; 2 UKCRC Centre of Excellence for Public Health (Northern Ireland), Institute of Clinical Science B, Royal Victoria Hospital, Belfast, Antrim, UK

## Abstract

**Introduction:**

Transient ischaemic attacks (TIAs) and strokes are highly prevalent conditions. Stroke killed 5.7 million people worldwide in 2005 and is estimated to cause 6.5 million deaths globally in 2015. Stroke survivors are often left with considerable disability. Many strokes are preceded by a TIA/‘minor’ stroke in the previous 90 days and therefore the immediate period after a TIA/minor’ stroke is a crucial time to intervene to tackle known vascular risk factors. Although rehabilitation following a TIA/minor stroke is widely recommended, there is a paucity of research that offers an evidence base on which the development or optimisation of interventions can be based, particularly for home-based approaches and non-pharmacological interventions in the acute period following the initial TIA/‘minor’ stroke. This systematic review will investigate the effect of rehabilitation programmes initiated within 90 days of the diagnosis of a TIA or ‘minor’ stroke aimed at reducing the subsequent risk of stroke.

**Methods/design:**

This systematic review will be reported in line with the Preferred Reporting Items for Systematic Reviews and Meta-analyses(PRISMA) guidance. Randomised and quasi-randomised controlled trials of rehabilitation programmes initiated within 90 days of a TIA or ‘minor’ stroke will be included. Articles will be identified through a comprehensive search of the following databases, guided by a medical librarian: the Cochrane Library, Web of Science, MEDLINE, Embase, CINAHL and PsycINFO. Two review authors will independently screen articles retrieved from the search for eligibility and extract relevant data on methodological issues. A narrative synthesis will be completed when there is insufficient data to permit a formal meta-analysis.

**Discussion:**

This review will be of value to clinicians and healthcare professionals working in TIA and stroke services as well as to general practitioners/family physicians who care for these patients in the community and to researchers involved in designing and evaluating rehabilitation interventions.

**Trial registration number:**

CRD42015016450.

## Background

### Stroke prevalence, impact and risk

Stroke killed 5.7 million people worldwide in 2005 and is estimated to cause 6.5 million deaths in 2015,^1^ with stroke survivors often being left with considerable disability.^2^ Many strokes are preceded by transient ischaemic attacks (TIAs) in the previous 90 days,^3^ and therefore the immediate period after a TIA is a crucial time to intervene to tackle the known vascular risk factors and reduce the risk of stroke. In 2006, approximately 1700 TIAs and 4000 strokes occurred in Northern Ireland alone.^4^

The 90-day risk of vascular events following a TIA or ‘minor’ stroke, excluding events within the first week after diagnosis when the risk is highest, can be as high as 18%.^5^ The ABCD^2^ score (consisting of age, blood pressure, clinical symptoms, duration of symptoms and presence of diabetes) in patients with TIA is used to identify the future risk of stroke.^3^ The presence of a new infarct, identified on brain imaging and indicating that the patient has actually had a stroke rather than a TIA, places the patient at higher risk of a further stroke within the first 90 days.^6^

### Secondary prevention reduces risk of second stroke

Immediate assessment of TIA and ‘minor’ stroke patients following the initial event, with initiation within 24 h of secondary prevention to tackle the known vascular risk factors, focused on pharmacological interventions, can reduce the 90-day risk of stroke to 2% within the research setting.^7^ Current guidelines however, recommend that patients should be assessed up to 1 week following a potential diagnosis,^8^ rather than within 24 h.^7^ Evidence is growing regarding the contribution of change in modifiable vascular risk factors to reductions in cardiovascular deaths and there is a need to consider how to promote non-pharmacological measures within secondary prevention.

### Underlying pathological mechanism and risk factors for TIA/'minor’ stroke

TIA and strokes are most commonly caused by the embolic or thrombotic consequences of atherothrombotic disease,^9^ which is similar to the underlying pathological mechanism for cardiovascular disease (CVD).^10–12^ As well as sharing a similar underlying pathological mechanism, cerebrovascular and CVD share common underlying risk factors^11^ ^13^ and there is a high prevalence of asymptomatic coronary artery disease post-TIA.^5^ ^12^ ^13–15^

The modifiable risk factors for all vascular diseases include smoking, excessive alcohol intake, physical inactivity, dietary factors, hypertension, dyslipidaemia, diabetes and obesity^16^ as well as low VO_2max_.^17–20^ Five modifiable risk factors are reported to account for 82% of strokes: hypertension, smoking, obesity, unhealthy diet and physical inactivity.^21^ Despite improvements in acute stroke care, prevention remains the cornerstone of reducing the stroke disease burden.^22^

### Why is it important to do this review?

Although rehabilitation and secondary prevention programmes following an initial TIA or ‘minor’ stroke are theoretically well-evidenced,^23^ ^24^ there is a paucity of well-informed rehabilitation programmes for clinicians and healthcare professionals to use within this acute period, particularly within the setting of the patient's home. This systematic review will help produce an evidence base on which rehabilitation interventions can be developed for this patient group, particularly within the acute period following the diagnosis of TIA or ‘minor’ stroke, including optimal pharmacology and non-pharmacological secondary vascular prevention.

## Aim

This systematic review will investigate the effect of rehabilitation programmes initiated within 90 days of a TIA or ‘minor’ stroke aimed at reducing the subsequent risk of stroke and specific vascular risk factors in adults.

### Key objectives

The key objectives of this study are to:

1. Determine the effect of rehabilitation programmes which have been initiated within 90 days of a patient having a TIA or ‘minor’ stroke, in terms of addressing the known vascular risk factors.
2. Determine the effect of rehabilitation programmes which have been initiated within 90 days of a patient having a TIA or ‘minor’ stroke, in terms of reduced risk of subsequent stroke.
3. Determine if particular techniques or components of programmes are associated with greater effect sizes.

## Methods/design

This review will be reported as per PRISMA guidelines (see online supplementary file 1). Criteria for considering studies for this review will include the below.

### Types of studies

All human randomised and quasi-randomised controlled trials, published and unpublished, aimed at initiating rehabilitation programmes within 90 days of patients suffering a TIA or minor stroke.

### Types of participants

The review will focus on adults aged 18 years or older, who have received a diagnosis of a TIA and/or ‘minor’ stroke, based on a clinical diagnosis, or on findings from brain imaging (eg, CT or MRI of the head). For clarity and to exclude more distinct populations, we will include only patients experiencing a TIA or ‘minor’ stroke. ‘Minor’ stroke will be defined by a score of three or less on the National Institutes of Health stroke scale at initial assessment as per previous authors^25^ or a modified Rankin scale of two or less. We will exclude moderate or severe patients with stroke. No restrictions will be made based on gender.

### Types of interventions

The WHO has defined rehabilitation as:

a set of measures that assist individuals who experience, or are likely to experience, disability to achieve and maintain optimal functioning in interaction with their environments^26^.

Any rehabilitation programme or intervention aimed at tackling secondary prevention of vascular events following a TIA or ‘minor’ stroke will be eligible for inclusion, for example, educational programmes, aerobic or exercise classes, self-management and lifestyle interventions. The review will include any 1:1 or group-based intervention, hospital-based, outpatient-based or home-based programmes. We will include trials with a comparative control group and trials with multiple intervention arms (comparing different types of rehabilitation interventions). The review will not include population or community-wide interventions (eg, mass media campaigns, built environment).

### Types of outcome measures

#### Primary outcomes

- Quantitative between-group differences for blood pressure, lipid profile (total cholesterol, high-density lipoprotein, low-density lipoprotein, triglycerides), glycaemic control in diabetes mellitus glycated hemoglobin, body mass index (BMI) or validated cardiovascular risk score.
- Any indicator of patient adherence to secondary prevention medications, for example, self-reported medication adherence or medication persistence, medication possession, individual patient data on prescriptions, pharmacy claims, electronic monitoring and drug tracers in the blood or urine.

#### Secondary outcomes

- Secondary cardiovascular events: stroke, myocardial infarction or vascular death.

### Search methods for identification of studies

To identify studies for inclusion in this review, detailed search strategies will be developed for each electronic database searched with input from a medical librarian. These will be based on the search strategy developed for Medical Literature Analysis and Retrieval System Online (MEDLINE) (see online supplementary additional file 1) but revised appropriately for each database.

### Electronic searches

We will search the Cochrane Central Register of Controlled Trials (CENTRAL) in the Cochrane Library, the Cochrane Database of Systematic Reviews (CDSR) in the Cochrane Library to December 2014, Ovid MEDLINE(R) Daily Update, Ovid MEDLINE(R) 1946 to December 2014, Ovid MEDLINE—includes new records not yet fully indexed, Ovid Embase 1974 to December 2014, EBSCO Cumulative Index to Nursing and Allied Health Literature (CINAHL) plus 1937 to December 2014 and Ovid PsycINFO 1806 to December 2014.

Indexed versions of Medline will be combined with the Cochrane Search Strategy for identifying randomised trials in MEDLINE: sensitivity-maximising and precision-maximising version (2008 revision).

CINAHL and Embase searches will be combined with the SIGN search filters developed to retrieve randomised controlled trials in these databases.

Any systematic reviews of rehabilitation interventions in the acute period following a TIA or ‘minor’ stroke will be screened for additional references. Additional studies will be identified from the reference lists of the retrieved papers. The reviewers will supplement the electronic search strategy by using the Science Citation Index to perform citation tracking of the trials identified by the first step.

### Study inclusion and exclusion criteria

The inclusion criteria are:

- Adults aged 18 years old or above who have been diagnosed with a TIA and/or ‘minor’ stroke.
- Assesses the impact of a rehabilitation programme initiated within 90 days of the diagnosis of a TIA or ‘minor’ stroke.
- The outcome measure for the study includes a cardiovascular risk factor (eg, blood pressure, physical activity levels), cardiovascular outcome (eg, further stroke event) and/or death.
- The study is a randomised controlled trial or quasi-randomised controlled trial.

The exclusion criteria are:

- Includes moderate and/or severe stroke subtypes.
- No rehabilitation service evaluated.
- No modifiable CVD risk factors, cardiovascular events and/or death outcomes reported.
- Protocol paper and therefore no results available.

### Data collection and analysis

#### Selection of studies

Results from the searches will be imported into EndNote (X7) bibliographic software (Thomson Reuters, Philadelphia, Pennsylvania, USA) and duplicates removed. The titles and abstracts of publications obtained by the search strategy will be independently screened by two authors (NH 100%, MEC 100%). Articles that do not meet the inclusion criteria will be removed. All remaining publications will be retrieved for further assessment. Based on the information within the full reports, two review authors (NH, MEC) will use a standardised form tested prior to use to select the trials eligible for inclusion in the review, if necessary, a third review author (FK) will resolve disagreements. A record will be kept of all articles excluded at this stage and the reason for their exclusion.

No language restrictions will be made; non-English papers will be assessed and, if necessary, translated with the assistance of a native speaker.

### Data extraction and management

Data will be extracted independently by two review authors (NH, MEC) using a customised form, piloted prior to use. This will be used to extract relevant data on methodological issues, eligibility criteria, interventions (including the number of participants treated, intervention provider) and study design, study duration, follow-up, comparisons, outcome measures, results, withdrawals and adverse events. The κ and percentage disagreement will be calculated, the two reviewers will meet to resolve any discrepancies, with third party adjudication if required.

In the case of multiple publications of the same study, the team will, where possible, extract and combine all of the available data, in case of doubt; the original publication will be given priority. Where data seem to be missing from a study this will, if possible, be obtained through correspondence with the study authors. A table showing the characteristics of the included and excluded studies will be created.

There will be no blinding to study author, institution or journal. Two authors will independently select studies for inclusion based on the following criteria. The study:

1. Restricted participants to TIA or ‘minor’ stroke patients, or reported outcomes separately for TIA or ‘minor’ stroke patient subgroups;
2. Evaluated a rehabilitation service intervention;
3. Stated or clearly implied that the intention of an intervention was to improve modifiable risk factor control and/or function;
4. Assessed one or more of the defined outcome measures.

The review team will resolve any disagreements regarding study eligibility by discussion between all review authors.

A record will be kept of the following information for each study:

1. General information: published or unpublished, title, authors, journal or source, publication date, country of origin, publication language.
2. Study methods: unit of randomisation (and method), allocation concealment (and method), blinding (outcome assessors), validation of questionnaires.
3. Participants: sampling (random or convenience), place of recruitment, total sample size, numbers randomised, inclusion criteria, exclusion criteria, demographic characteristics (age, gender, ethnicity, socioeconomic or sociodemographic status), disability (modified Rankin score, Barthel score), comorbidities, similarity between groups at baseline, dropout and withdrawal rates.
4. Intervention details: components, length, frequency, location, mode of delivery, personnel responsible for delivery, timing post-stroke, details of control protocol.
5. Outcomes: prespecified outcomes defined above, follow-up intervals from start of intervention, units of measurement, missing data.
6. Results: results for prespecified outcomes, number of participants assessed, method of analysis (intention-to-treat analysis, per protocol analysis).
7. Intervention category: prespecified in the review protocol.

### Assessment of risk of bias

Two review authors (NH, MEC) will independently assess each included study for risk of bias using the risk of bias tool, following guidance from the Cochrane Handbook of Systematic Reviews of Interventions.^27^ The following domains will be considered:

- Was the allocation sequence adequately generated?
- Was the allocation adequately concealed?
- Was knowledge of the allocated intervention adequately prevented during the study?
- Were incomplete outcome data adequately addressed?
- Are reports of the study free of suggestion of selective outcome reporting?
- Was the study apparently free of other problems that could put it at a high risk of bias?

To minimise bias in interpretation of the tool, a small sample of unrelated studies will be assessed. Inconsistency in scoring will be reviewed, and a consensus reached prior to the analysis of the review studies. The tool will be used to judge and report whether a trial is deemed to be at ‘high’, ‘low’ or ‘uncertain’ risk of bias. A summary statement regarding the quality of the data included in the review and a narrative account of any serious flaws will be reported.

#### External validity

To ensure the results of the systematic review and meta-analysis are generalisable to the true study population of interest (those with a TIA and'or ‘minor’ stroke diagnosis) we will consider the external validity of all included studies. To do this we will report on relevant aspects of the included studies in the review,^28^ including:

- Participant characteristics with reference to the background population (age, gender, source of recruitment);
- Sample size;
- Type and characteristics of the rehabilitation intervention.

This will allow us to comment on how representative the included studies are of the intended true population and therefore allow clinicians to better apply the evidence to their study population of interest.

### Measures of treatment effect

For each study, relative risk and 95% CIs will be calculated for dichotomous outcomes, and mean differences and 95% CIs will be calculated for continuous outcomes. Where continuous outcomes are pooled on different scales, standardised mean differences will be used. Where available, changes from baseline (mean change scores) will be used in preference to follow-up scores.

### Unit of analysis issues

The review team anticipates two possible units of analysis issues that may arise; repeated observations of the same outcome and studies including multiple intervention arms.

If studies report multiple observations of the same outcome, the reviewers will extract data at the following time points: baseline, short-term (not longer than 12 weeks post-randomisation), medium-term (not longer than 6 months postrandomisation), and long-term (greater than 6 months postrandomisation) follow-up.

In the case of studies including multiple intervention groups, the reviewers will follow the recommended method suggested by the Cochrane Collaboration section 16.5^27^ for combining multiple groups from one study. For continuous outcomes, means and SDs will be combined using methods and formulae described in chapter 7 (7.7.3.8)—combining groups, in the Cochrane Handbook of Systematic Reviews of Interventions.^27^

The following will be considered in relation to assessing the risk of bias in multiple intervention studies:

- Are data presented for each of the groups to which participants were randomised?
- Are reports of the study free of suggestion of selective reporting of comparisons of intervention arms for some outcomes?

### Missing data

Attempts will be made where necessary to contact original investigators to request missing data. If SDs are missing from continuous data, studies will be scanned for other statistics such as CIs, SEs or p values that would allow for its calculation. If there are a large number of missing SDs, then imputation will not be carried out.

### Assessing for heterogeneity

Diversity across the studies will be assessed qualitatively in terms of intervention (content, duration, frequency, provider and setting), participant demographics, outcome measures and follow-up. If two or more studies are considered clinically homogenous according to the above terms, data will be assessed for statistical heterogeneity using RevMan V.5.1. The review team will use the χ2 test in conjunction with the I^2^ statistic. The level of significance for the χ2 will be set at p<0.1. Values of I^2^ that are 30–60% will be considered to represent moderate heterogeneity and 50–90% substantial heterogeneity.^27^ In the case of substantial heterogeneity, the review team will pool studies using a random effects model; in the case of low or no heterogeneity, the review team will analyse studies using a fixed effects model. A sensitivity analysis will be performed to investigate the effect of inclusion and exclusion of heterogeneous studies.

### Assessment of reporting bias

A funnel plot will be prepared if there are sufficient studies by plotting trial effect against SE. The plot will be inspected for asymmetry to investigate reporting bias.

### Data synthesis

In complex healthcare interventions, effects can be modified by a wide variety of factors and we anticipate a high degree of heterogeneity within the included studies. Careful consideration will be given to the appropriateness of conducting a meta-analysis. Data will be summarised statistically when the data are available, sufficiently similar and of sufficient quality. The statistical analysis will be performed in accordance with the statistical guidelines referenced in V.5.1.0 of the Cochrane Handbook for Systematic Reviews of Interventions.^27^

A narrative synthesis will be completed if there is insufficient data to permit a formal meta-analysis. The narrative synthesis will attempt to summarise the current state of knowledge, describe the interventions, study designs and the robustness of the evidence.

### Subgroup analysis and investigation of heterogeneity

Outcome assessment data for all time periods where available will be grouped into three time periods for the purposes of analysis: baseline (0–3 months), medium-term (3–6 months), and long-term follow-up (greater than 6 months).

Where possible, the following subgroup analysis will be performed:

- Duration and/or frequency of intervention (previous reviews have noted correlations between effect and duration of interventions).
- Recruitment setting—that is, primary or secondary care
- Initial clinical conditions (TIA or ‘minor’ stroke).
- Whether the intervention is hospital-based, community-based or home-based.

### Sensitivity analysis

A sensitivity analysis may be performed to check if including or excluding studies of lower methodological rigour or higher risk of bias affects the comparison between groups. If sensitivity analysis appears to influence the findings of the review, this will be reported in the ‘Discussion’ section.

## Discussion

Rehabilitation programmes following an initial diagnosis of a TIA or ‘minor’ stroke have the potential to address the increased risk of morbidity and mortality in this population. Reviews identifying effective components of rehabilitation following the diagnosis of a stroke have begun to emerge,^23^ ^24^ but to date, this will be the first systematic review to review the use of rehabilitation programmes in the acute period (within 90 days) of the diagnosis of a TIA or ‘minor’ stroke. The findings of this review may be applied in clinical and rehabilitation settings but will also be of value to those involved in designing and developing complex healthcare interventions.
